# Acute Abdomen Is a Rare Presentation of COVID-19 in the Absence of Respiratory Manifestations: A Case Report

**DOI:** 10.7759/cureus.42518

**Published:** 2023-07-26

**Authors:** Abbas A Mohamed, Mohammed Alharbi, Sarah A Mohamed

**Affiliations:** 1 Department of General Surgery, King Salman Specialty Hospital, Hail, SAU; 2 Department of Surgery, Al Imam Mohammad Ibn Saud Islamic University, Riyadh, SAU; 3 Department of General Surgery, University Hospital of Wales, Cardiff, GBR

**Keywords:** conservative management, perforated duodenal ulcer, acute abdomen, pandemic, corona virus

## Abstract

Mild gastrointestinal symptoms and mild abdominal pain often occur in association with COVID-19. However, acute abdomen and severe abdominal pain warranting urgent surgical treatment are rare. Here we present the case of a 40-year-old man who presented with the clinical picture of a perforated duodenal ulcer. He was eventually found to have COVID-19 and was treated conservatively. In this report, we discuss his course of treatment and review the relevant literature.

## Introduction

Novel coronavirus disease (COVID-19) caused by severe acute respiratory syndrome coronavirus 2 (SARS-CoV-2) was first described in the Hubei region of China in 2019 and declared a pandemic by the World Health Organization (WHO) in March 2020 [1]. COVID-19 is primarily a respiratory virus, affecting mainly the lungs. Mild gastrointestinal (GI) symptoms such as nausea, vomiting, loss of appetite, and diarrhea were observed in the initial phase of the pandemic; acute severe GI illness was rarely reported in association with the infection. Surgical assessment of positive COVID-19 with acute abdomen can be challenging, but rewarding, as it can avoid unnecessary surgery. Here we report a rare case of acute abdomen due to COVID-19 mimicking a perforated duodenal ulcer.

## Case presentation

**Figure 1:**
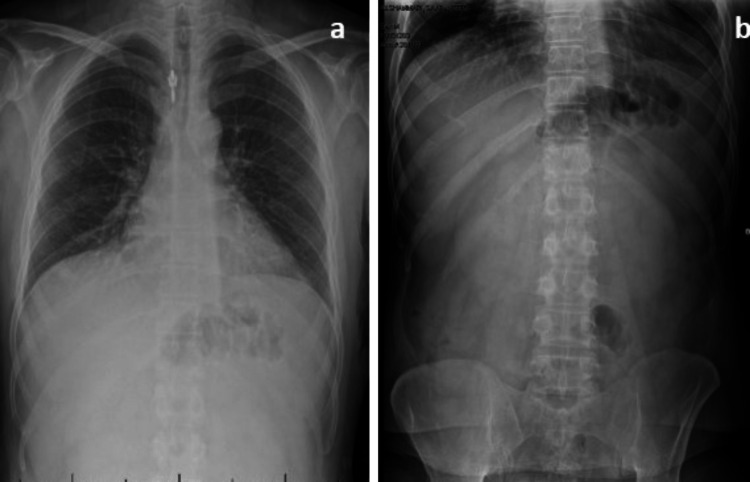
X-ray of the chest in the upright position (a) and X-ray of the abdomen (b) showing no air under the diaphragm.

A 40-year-old Saudi man was admitted to our emergency department with a sudden onset of severe epigastric pain associated with frequent vomiting. The pain had occurred after three days of flu-like symptoms for which he had not seen a doctor. He was otherwise healthy and had no chronic illnesses apart from recurrent dyspepsia, which usually resolved with antacids, and he had never had surgery. On examination, the patient looked ill and had tachycardia (pulse of 110 beats per minute), blood pressure of 128/82 mm Hg, fever (38.6°C), respiratory rate, and oxygen saturation were normal. Chest examination revealed no basal crepitations or wheezing on either side of the chest. Abdomen examination revealed severe tenderness and board-like muscle rigidity over the epigastrium. The rest of the abdomen was lax and not painful. Bowel sounds were audible. Laboratory investigations revealed a leukocyte count of 11.2 (109/liter), and a normal hemoglobin of 13.6 g/L. Serum creatinine was slightly elevated (136 mmol/L), blood urea and electrolyte levels, and liver function profile were within normal range. Pancreatic enzymes were within the normal range. The screening test for COVID-19 was initially negative. The C-reactive protein was 108 mg/L. Given the history of chronic dyspepsia, the sudden onset of severe epigastric pain, and the abdominal examination findings, a diagnosis of perforated peptic ulcer was made, but the X-ray of the abdomen and thorax in the upright position showed no air under the diaphragm (Figures 1a-1b).

**Figure 2:**
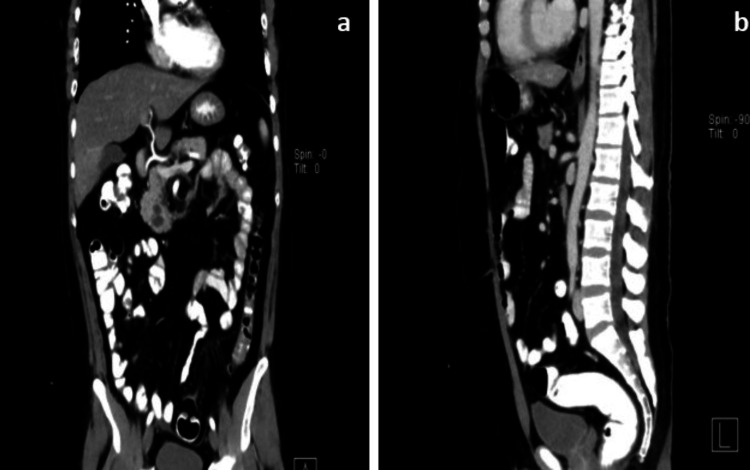
A double-contrast CT scan image. (a) Sagittal section and (b) coronal section, both showing no free air in the abdomen and no intraperitoneal leakage of oral contrast medium. CT: computed tomography

For further examination, a double contrast scan computed tomography (CT) was requested, which showed no free air in the abdomen and no intraperitoneal leakage of the oral contrast medium (Figure 2). A chest examination CT was not performed because the lung field was clear on the chest X-ray, but the lower parts of the chest on the abdominal CT scan showed no lung infiltration.

Considering the result of the radiological examination, we planned to treat the patient conservatively. He was admitted to the surgical ward, kept fasting, and given an intravenous antibiotic (cefuroxime) and intravenous fluids. The next day, the polymerase chain reaction (PCR) of nasopharyngeal swabs was positive for COVID-19. The patient was isolated and given permission for oral intake and continued antibiotics, resulting in a dramatic clinical response. He was discharged five days after admission.

## Discussion

COVID-19 caused by severe acute respiratory syndrome coronavirus 2 (SARS-CoV-2) was first described in the Hubei region of China in 2019 and declared a pandemic by the World Health Organization (WHO) in March 2020 [1].

COVID-19 is primarily a respiratory disease affecting the lung parenchyma, but it may also affect several organs and cause various extrapulmonary symptoms. As the disease progressed, more and more reports of extrapulmonary disease emerged, reporting cardiovascular, GI, renal, neurological, and skeletal-muscular involvement, either in isolation or in association with a pulmonary manifestation of the disease.

COVID-19 is primarily a respiratory infection causing fever, cough, shortness of breath, and loss of smell or taste. However, mild GI symptoms such as nausea, vomiting, and diarrhea have been reported in association with pulmonary manifestations of the disease, but acute abdomen requiring immediate life-saving surgical treatment has rarely been reported in association with the infection.

The exact mechanism of how COVID-19 causes GI symptoms and acute abdomen is not yet fully understood. Still, some possible explanations include infection of the cells of the GI tract by binding of the virus to the angiotensin-converting enzyme 2 (ACE2) receptor expressed in the esophagus, stomach, small and large intestine, inflammation and damage to the mucous lining, and the triggering of an excessive immune response (cytokine storm) that can cause systemic inflammation and multi-organ failure and lead to ischemia and necrosis of the intestine or other abdominal organs, and the causation of coagulation abnormalities that can increase the risk of thrombosis in the mesenteric vessels and lead to intestinal ischemia and infarction.

Safari et al. suggest that COVID-19 uses ACE2 as an entry receptor to interact with host cells. Therefore, tissues with high ACE2 expression, such as lungs and visceral adipose tissue, could be a potential target for this new virus [2]. Zhao D et al. proved that the epithelium of the stomach, duodenum, and rectum exhibit strong staining of this receptor [3].

The positive correlation between COVID-19 and the incidence of peptic ulcers was suggested by the study of Dao et al., which showed that the incidence of peptic ulcers was significantly higher during the COVID-19 pandemic in 2020 compared to 2019 in the same health facility in Vietnam [4], and by the study of Jian et al., which confirmed the same findings in two hospitals in Wuhan, China, during the pandemic [5].

A meta-analysis of 35 Chinese studies involving 6686 patients with COVID-19 found a pooled prevalence of digestive symptoms of 15%, with nausea or vomiting, diarrhea, and loss of appetite being the three most common symptoms. There was also a correlation between the presence of GI manifestations and clinical deterioration [6,7], while an Italian study of 292 patients found a high prevalence of GI symptoms (diarrhea and vomiting) in COVID-19 patients. Interestingly, the presence of GI manifestations is associated with a better prognosis [6,8].

Melazzini et al. reported five patients with GI symptoms without respiratory symptoms. All had positive PCR for COVID-19 and were diagnosed with peptic ulcer on admission with no history of peptic ulcer or Helicobacter pylori infection. They suggested that the pathogenesis of peptic ulcer disease could be explained by stress due to fear of a pandemic or acute illness, by direct damage to the gastric epithelium by the virus, or by active inflammation of the mucosa maintained by the cytokine storm [9].

Acute abdomen is a rapid onset of severe symptoms that may indicate life-threatening intra-abdominal pathology requiring urgent attention and treatment [10]. Common causes of acute abdomen are acute appendicitis, cholecystitis, pancreatitis, diverticulitis, and acute peritonitis.

Diagnosis of COVID-19 with acute abdomen can be difficult as symptoms are non-specific and may mimic other causes of abdominal pain. The rare occurrence of COVID-19 as acute abdomen, especially in the absence of respiratory manifestations of the disease, leads to unnecessary harmful surgical interventions. In addition, surgical complications of GI disease such as perforations can be difficult to diagnose in critically ill, intubated patients, leading to misdiagnosis of severe cases requiring surgical intervention.

The assessment of COVID-19-positive patients with acute abdomen and the decision to operate should be made with great caution, as unnecessary surgery in such patients may lead to a cytokine storm and worsen the patient’s condition. An active observation protocol with repeated clinical examination and repeated determination of inflammatory markers in the blood is more useful than a hasty negative exploration.

There are several reports on the association of COVID-19 with the non-complicated peptic disease, but unfortunately, there are not a large number of published studies with large samples on this topic, probably because of the rarity of the association between COVID-19 and acute abdomen. Most of the available literature consists of sporadic case reports. To our knowledge, our case is the first case reported in which severe symptoms and signs of peptic ulcer perforation were the main features of the disease, without respiratory symptoms, and actual perforation.

Merdad GA et al. reported a case of COVID-19 with classical complaints suggestive of complicated peptic ulcer disease without respiratory symptoms. After laboratory and radiographic investigations, the patient was diagnosed with a perforated duodenal ulcer and underwent laparotomy. The patient had a complicated postoperative course [11].

Similarly, He L et al. reported the case of a COVID-19-positive patient with lung involvement on CT. Five days after admission, he developed clinical and radiological signs of a perforated duodenal ulcer. The patient underwent surgery and was successfully treated [12].

Agnes A et al. reported on a COVID-19-positive patient with severe respiratory syndrome who was treated with multiple doses of IL-6 inhibitors and underwent successful emergency surgery for a perforated duodenal ulcer. They concluded that the association between repeated administration of IL-6 inhibitors and upper GI bleeding and perforation needs to be investigated and that the threshold for administration of prophylactic therapy with proton pump inhibitors in patients with severe COVID-19 should be carefully considered [13].

## Conclusions

COVID-19 is primarily a respiratory infection. However, mild GI symptoms such as nausea, vomiting, and diarrhea have been reported in association with the disease. Acute abdomen requiring immediate life-saving surgical decisions and treatment has rarely been reported in association with the infection. Diagnosis of acute abdomen in COVID-19 patients can be difficult as the symptoms may represent the main symptoms of the disease without intra-abdominal pathology being present. The situation is even more deceptive in the absence of respiratory symptoms of the disease and may lead to unfavorable surgical intervention. The assessment of COVID-19-positive patients with symptoms and signs of acute abdomen and the decision to proceed with surgery should be made with great caution, as surgery in these patients may lead to a cytokine storm and worsen the patient’s condition.
